# Prevalence, pattern and impact of sleep disturbance on quality of life and exercise participation among children with cerebral palsy in Kano city

**DOI:** 10.5935/1984-0063.20200108

**Published:** 2021

**Authors:** Umaru M. Badaru, Aliya M. Hassan, Rufai Y. Ahmad, Jibril M. Nuhu, Isa U. Lawal

**Affiliations:** 1 Bayero University, Kano, Physiotherapy - Kano - Kano - Nigeria.; 2 Federal Medical Center, Physiotherapy -Azare - Bauchi - Nigeria.

**Keywords:** Exercise, Quality of Life, Cerebral Palsy, Sleep Deprivation

## Abstract

**Introduction:**

Sleep disturbance (SD) could have negative impact on the general well-being of children with cerebral palsy (CWCP).

**Objectives:**

The purpose of this study was to assess the prevalence of SD and its impact on quality of life and exercise participation among CWCP.

**Material and Methods:**

In the cross-sectional study, CWCP and their siblings were recruited from secondary and tertiary hospitals in Kano City. SD, gross motor function (GMF), spasticity and quality of life were assessed with SD scale, GMF classification system, modified Ashworth scale and pediatric quality of life inventory, respectively. Data was analyzed with Mann-Whitney U and chi-square tests, linear and hierarchical regressions using SPSS version 20.0.

**Results:**

There were 200 CWCP (aged 4.35±8.03 years) and 200 siblings (aged 5.89±3.06 years). The prevalence of SD in CWCP was 31.5%. CWCP suffered more SD than their siblings (p<0.001). SD in CWCP is influenced by GMF level (ß=0.378, p<0.001) and gender (ß=0.16, p<0.05). SD has negative influence on quality of life (ß=-0.18, p<0.001), active participation in home-based (ß=-0.23, p<0.000), and clinic-based exercises (ß=-0.24, p<0.00). GMF levels (ß=-0.505, p<0.0001), hamstring spasticity (ß=-0.250, p<0.005), and age (ß=-0.207, p<0.001) also have influenced on quality of life.

**Conclusion:**

One-third of the CWCP suffered pathologic SD, which has negative impact on their quality of life and the ability to actively participate in both home and clinic-based exercises. Aside SD, other factors such as child’s age, spasticity level and severity of motor impairment also affected their quality of life negatively. Enhancing the motor abilities of CWCP may improve their quality of sleep and quality of life.

## INTRODUCTION

Cerebral palsy (CP) is a common cause of physical disability among children worldwide. In addition to motor impairment that is the hallmark of C P, sleep disturbance (SD) could lead to deterioration of their physical functioning and quality of life^1^. Researches have reported that SD is common in children with CP (CWCP)^2, 3, 4, 5, 6^. It has also been reported that SD is more commonly found in CWCP when compared with typically developing children^4, 6, 7, 8^. Impaired mobility^9, 10^, pain^9, 11^, seizures and epilepsy^8, 9, 10^, and poor body positioning^11^ were reported to have negative impact on the quality of sleep in CWCP.

Often, rehabilitation specialists tend to be more concerned about improving the functional activities that are carried out by the CWCP in the daytime, and are likely to overlook nighttime activities such as quality of sleep^5, 12^. It has been opined that SD can impact negatively on the daily functioning of a child^13, 14^. Excessive daytime sleepiness may interfere with active participation of CWCP in both clinic and home-based exercises. The prevalence of SD in CWCP has been reported in Europe^2, 7^, Malaysia^4^, and Uganda^10^. There is, however, a dearth of studies from Nigeria to highlight the magnitude of SD in CWCP.

Several studies have reported that CP is associated with deterioration of children’s quality of life^15, 16, 17, 18, 19^. Furthermore, findings from studies^20, 21^ have also shown that SD has negative impact on quality of life in CWCP. It was reported that excessive daytime sleepiness, insomnia^3^, and altered sleep pattern^20^ were associated with reduced quality of life. Studies are however yet to clarify whether the impairment of quality of life in CWCP is actually influenced by SD alone owing to the fact that the other factors such as pain^16, 17^, age^18, 19^, severity of motor disability^18^, and impairments of intellectual functioning^16^, could also lead to the deterioration of their quality of life.

Sleep is an essential physiological process, and good quality sleep is essential for all humans to achieve overall health for the execution of normal daily activities. Sleep anomaly due to a primary or secondary cause can profoundly impact on quality of life, and CWCP who experience SD often present with compromised quality of life. The outcome of any research endeavour that seeks to investigate the influence of SD on quality of life in CWCP may not necessarily suggest the impact of SD alone on quality of life. In connection with the aforementioned, it is important to consider other factors such as type and severity of CP, medications that are routinely administered to these children, age, gender, spasticity, and epilepsy which may likely contribute to decrease in QOL. We also argue that in spite of the adverse effect SD may have on the level of physical functioning of a child, physiotherapists managing CWCP seldom assess and report its influence on the level of children’s participation in both clinic and home-based exercise. Finally, there is a dearth of studies highlighting the possible impact SD may have on the level of exercise participation in CWCP. This study therefore assessed the prevalence of SD and its impact on quality of life and exercise participation among CWCP.

## MATERIAL AND METHODS

It was a cross-sectional study in which CWCP and their siblings were recruited from Aminu Kano Teaching hospital, Murtala Muhammad Specialist Hospital, Hasiya Bayero Paediatric Hospital and Abdullahi Wase Specialist Hospital, all in Kano city. Ethical approval for the study was obtained from ethics committees of Aminu Kano Teaching Hospital (NHREC/21/08/2008/AKTH/EC/2563) and that of Kano State Ministry of Health (MOH/OFF/797/ TI./1505). Excluded are children with severe chest infections with coughs or severe musculoskeletal pain due to injury that may cause SD. Written informed consent was sought and obtained from each of the caregivers after the study procedure has been explained to them. CWCP and their typically developing siblings were recruited from the outpatient units of physiotherapy departments of the selected hospitals.

### Sample size determination

CP has a prevalence of 0.106 per 1,000 in Kano State . The prevalence of 0.11 was used to calculate sample size for the study using the sample size formula for prevalence studies.


$$N=\frac{Z^{2}P{\left(1-P\right)}^{23}}{d^{2}}$$

N=Sample size;

P=prevalence

Z is a constant=1.96 at 95% Confidence Interval, d= precision=0.05 N=(1.96)^2^x0.11(1-0.11)/(0.05)^2^=3.842x0.098/0.0025=151 participants.

### Data collection procedure

A data capture form was used to record characteristics of the children. Motor function was assessed with gross motor function classification system (GMFCS). The GMFCS is a 5-level ordinal scale scored from I to V , CWCP in GMFCS levels I-III are ambulant without much restriction of independent mobility. CWCP in level III walks with walking aid. Children in levels IV and V are non-ambulant with severe restriction of independent mobility. Some CWCP in level IV can use powered mobility. All the participants were evaluated for presence of spasticity using the modified Ashworth scale (MAS). The MAS is a 6 point ordinal scale scored from 0 to

5. A score of 0 means no spasticity and the score of 5 means the limb is rigid in either flexion or extension. The presence of spasticity was indicated by a catch or resistance to passive stretching of muscle when patient is fully relaxed. Spasticity was assessed in biceps, hamstrings, and hip adductors muscles because majority of the CWCP in this study especially those with severe CP who are non-ambulant presented with bent knees and elbows with marked spasticity of the hamstrings, biceps, and hip adductor muscles. All spasticity measurements were conducted in the morning between 9-11 a.m. All participants with confirmed diagnosis of epilepsy by a physician were captured as having epilepsy in the proforma.

### Assessment of SD

This was assessed using SD scale. It has good internal consistency Cronbach’s-alpha=0.81^24^ and test-retest reliability r=0.71^25^. The instrument contains 6 domains, 26 items and each of the items was rated on a 5 point scale^25^. Sum of scores of the 6 domains yields a total sleep score^7, 26^. A T-score >70 that corresponds to a raw score of 52 or higher in the scoring sheet was rated as pathologic SD^7, 10, 26, 27^. Raw scores less than 52 (or T-score ≤70) were rated as insignificant SD.

### Assessment of quality of life

Quality of life was assessed with the parent version of pediatric quality of life inventory (CP-module). The questionnaire has high internal consistency of α=0.91^28^ and α=0.96^29^. It consists of 35-items and seven domains. However, the version of the questionnaire for toddlers (aged 2-4 years), consists of 22 items and 6 domains. It enquires about how much of a problem each item has been during the past 1 month. The questionnaire was scored on a 5-point ordinal scale from 0 = “never a problem” to 4 = “almost always a problem”. Items were reverse scored and linearly transformed to a 0-100 scale (0=100, 1=75, 2=50, 3=25, 4=0). The mean scores are computed as the sum of the items divided by the number of items answered so that higher scores indicated better quality of life.

### Assessment of perceived active participation in home and clinic-based exercise among CWCP

An 8-item questionnaire was designed to elicit responses from the caregivers whether their children experience problem with sleep and how often does it affect the children’s ability to actively participate in clinic and home-based exercise. The internal consistency of the questionnaire assessed on 30% of the study participants was α=0.72 and test retest reliability ICC=0.97. Items 2 and 3 (each scored on a scale of 1-4) were used to rate the level of active participation of CWCP during home and clinic-based exercises, respectively, in the last one month while questions 5 and 6 (each scored on a scale of 1-5) were used to rate number of times in the last one month that problem with sleep prevented the children from participating in home and clinic-based exercises, respectively. The sum of the scores of items 2 and 5 gave the level of perceived participation in home-based exercise and sum of the scores of items 3 and 6 gave the level of perceived participation in clinic-based exercise. The scores of items 5 and 6 were reversed coded such that CWCP who never had problem with sleep interfering with exercise have the highest scores, while those who had such problems almost all the time have the least scores. The highest perceived participation score is 9 (4+5) and the lowest score is 2 (1+1) for each of home and clinic-based exercises, the higher the score, the better the perceived participation.

### Data analysis

Data was summarized with descriptive statistics. Man-Whitney U test was used to assess the difference in SD between CWCP and their typically developing siblings; and the difference in quality of life between CWCP who had pathologic SD and those without it. Spearman’s rank order correlation and chi-square tests were used to find correlation and association, respectively, between SD and each of clinical and demographic characteristics of CWCP. Linear and hierarchical regressions were used to determine the variables influencing SD, quality of life, and exercise participation. Analysis was performed using SPSS version 20.0 at *p*<0.05.

## RESULTS

### Characteristics of CWCP

A total of 200 CWCP aged 1-15 years; mean age 4.34±2.78 years participated in the study. There were 118 (59%) males and 82 (40.8%) females. Their age categories were: 1-5 years 151 (75.5%), 6-10 years 42 (21%), and >10years 7 (3.5%). Their GMFCS categories were: I = 2 (1%), II = 27(13.5%), III = 53 (26.5%), IV = 35 (17.5%), and V = 83 (41.5%). Spastic CP types include quadriplegia 69 (34.5%), diplegia 11 (5.5%), hemiplegia 86 (43%), and monoplegia 5 (2.5%); while those with extrapyramidal CP mainly had athetosis 29 (14.5%). About 57 (28.5%) were diagnosed with epilepsy and 143 (71.5%) have no epilepsy. Majority of them 160 (80%) are underweight (<18kg/m^2^) and few 40 (20.0%) have normal weight (18-25kg/m^2^). Most of the CWCP have been placed on medications 117 (58.5%) with encephabol being the frequently prescribed drug 59 (50.43%) as presented in Table 1.

**Table 1:** Common medications prescribed to the study participants.

Name of Drug	Indication	Side Effects	Frequency (Percent)
Phenobarbitone			10 (8.55)
Phenobarbitone + Encephabol	Anticonvulsant	Sedation and hypnosis	9 (7.69)
Phenobarbitone + Carbamazepine			2(1.71)
Carbamazepine			7 (5.98)
Carbamazepine + Baclofen	Anticonvulsant	Sleepiness and drowsiness	1(0.86)
Carbamazepine + Encephabol			5(4.27)
Encephabol (Pyritinol)			59(50.43)
Encephabol + diazepam	Nootropic, CNS activating	Insomnia	1(0.86)
Encephabol + baclofen			1(0.86)
Valproic Acid (VA)			10(8.55)
VA + Baclofen VA + diazepam	anticonvulsant	Sleepiness, hypnosis and dry mouth	1(0.86) 1(0.86)
VA + Encephabol			4(3.42)
Baclofen	Antispastic	Sedation	4(3.42)
Diazepam	Antispastic	Sleepiness	1(0.86)
Benztropine	Anticholinergic/Anti-spasm	Drowsiness, dizziness and Dry mouth	1(0.86)
		Total	117(100)

CNS = central nervous system

### Characteristics of the siblings

A total of 200 siblings participated in the study with a mean age of 5.89±3.06 years (range 1-18 years). Majority of the siblings 118 (59.0%) are males and 82 (41.0%) are females.

### Prevalence and pattern of SD in CWCP and their siblings

In this study, 137 (68%) of the CWCP have insignificant SD while 63 (31.5%) have pathologic SD. The prevalence of pathologic SD among CWCP in this study was 31.5%. The overall mean score of SD for CWCP and their sibling were 45.18±9.55 and 34±5.35, respectively. There was a significant difference in the overall score of SD between CWCP and their siblings (*p*=0.001) (Table 2). CWCP suffered significantly from disorders excessive somnolence 59 (29.5%), sleep wake transition 42 (21.0%) and that of initiation and maintaining sleep 42 (21.0%). Pathologic sleep breathing disorders were also frequent 33 (16.5%) as presented in Table 2.

**Table 2:** Comparison of SD between CWCP and their Typically Developing Siblings.

Variables	CWCP Mean ± SD	SIBLINGS Mean ± SD	U-value	Z-value	P-value
Overall SD score	45.18±9.55	34±5.35	6447	-11.73	0.001*
Frequency of pathologic SD	63(31.5)	0(0)			
Disorders of initiation and maintaining sleep	12.51 ± 4.35	8.47 ± 1.52	7581.50	-10.87	0.000*
Frequency of pathologic SD	42(21%)	0(0%)			
Sleep breathing disorders	4.22 ± 1.86	3.54 ± 1.12	16270.50	-3.93	0.000*
Frequency of pathologic SD	33(16.5%)	11(16.5%)			
Disorders of arousal	3.16 ± 0.57	3.29 ± 1.14	19853.00	-0.267	0.790
Frequency of pathologic SD	4(2%)	7(3.5%)			
Sleep wake transition disorders	10.92 ± 3.36	8.52 ± 2.20	11427.00	-7.51	0.000*
Frequency of pathologic SD	42(21%)	5(2.5%)			
Disorders of excessive somnolence	10.27 ± 3.89	6.67 ± 2.02	8261.50	-10.28	0.000*
Frequency of pathologic SD	59(29.5%)	2(1%)			
Sleep hyperhidrosis	4.24 ± 2.03	3.65 ± 1.87	16434.50	-3.18	0.001*
Frequency of pathologic SD	18(9%)	13(6.5%)			

*significant; SD=sleep disturbance

In this study, there was a significant weak positive correlation between SD and gross motor function (GMF) levels (Rho=0.37, *p*=0.00). But there were no significant correlations between SD and spasticity levels in the hamstrings (Rho=0.07, *p*=0.36), biceps (Rho=0.11, *p*=0.14), and hip adductor muscles (Rho=0.08, *p*=0.25). There were significant associations between SD in CWCP and each of epilepsy (χ^2^=7.36, *p*=0.01) and medication use (χ^2^=7.98, *p*=0.001). There were however no significant associations between SD and each of age category (χ^2^=0.03, *p*=0.88), gender (χ^2^=0.45, *p*=0.50), and type of CP (χ^2^=3.45, *p*=0.49) as presented in Table 3.

**Table 3:** Association between SD and each of medication use, clinical and demographic characteristics of the CWCP.

Variables	SD category		Total (%)	χ^2^	P-value
	Insignificant SD (%)	Pathologic SD (%)			
**Gender**					
Males	83(60.6)	35(55.6)	118(59.0)	0.451	0.502
Females	54(39.4)	28(44.4)	82(41.0)		
**Age category**					
<4years	70(51.1)	33(52.4)	103(51.5)	0.029	0.880
≥4years	67(48.9)	30(47.6)	97(48.5)		
**Epilepsy**					
Yes	31(22.6)	26(41.3)	57(28.5)	7.360	0.007*
No	106(77.4)	37(58.7)	143(71.5)		
**CP Type Spastic (pyramidal)**					
Quadriplegia	46(33.6)	23(36.5)	69(34.5)		
Diplegia	7(5.1)	4(6.3)	11(5.5)	3.452	0.485
Hemiplegia	57(41.6)	29(46.0)	86(43.0)		
Monoplegia	5(3.6)	0(0)	5(2.5)		
**Non-spastic (Ex-pyr) Medication use**	22(16.1)	7(11.1)	29(14.5)		
No	66(48.2)	17(27.0)	83(41.5)	7.982	0.005*
Yes	71(51.8)	46(73.0)	117(58.5)		

Key: *significant level, χ^2^ =chi square value, P=probability value, CP=cerebral palsy, Exra- pyramidal

Linear regression analysis revealed that the most important factors influencing SD in CWCP were GMF level (β=0.378, *p*=0.00) and gender (β=0.16, *p*=0.024) R=0.44 or 44%, F-ratio=4.53 *p*=0.000 as shown in Table 4.

**Table 4:** Factors influencing SD among CWCP.

Variables	Standardized coefficient (β)	P-value
GMFCS	0.39	0.000*
Medication	0.09	0.21
Age	-0.02	0.78
Gender	0.15	0.02*
BMI	0.05	0.56
Type of CP	-0.03	0.78
Hamstrings spasticity	-0.04	0.75
Biceps spasticity	0.00	0.99
Hip adductor spasticity	-0.04	0.73
Epilepsy	-0.13	0.08

### Correlation between SD and quality of life of CWCP

There was a significant negative correlation between SD and quality of life (Rho=-0.37, *p*=0.001).

### Comparison of quality of life between CWCP having insignificant SD and those with pathologic SD

Furthermore, the mean score of quality of life in CWCP who have insignificant SD (N=137) was 56.55±15.70, while the mean score for those with pathologic SD (N=63) was 43.67±13.67.

CWCP who had pathologic SD have significantly lower quality of life when compared with those having negligible SD (U=2796.50; Z-value=-3.996, *p*<0.001).

### Factors influencing the quality of life of CWCP

Hierarchical regression analysis revealed that SD has significant negative impact on the quality of life of CWCP (β=-0.177, *p*=0.001). This study, however, observed that other clinical variables such as the GMF levels (β=-0.505, *p*=0.000), hamstring spasticity (β=-0.250, *p*=0.008), and age (β=-0.207, *p*=0.001) also have significant influence on quality of life even after control as presented in Table 5.

**Table 5:** Factors Influencing the Quality of Life of CWCP.

	Variables	P-Value model 1	P-Value model 1	Standardized Coefficient Beta Model 2	R square	R square Change	F-Value	P-value
	Age	0.001*	0.001*	-0.207				
	Gender	0.538	0.279	0.055				
	BM1	0.735	0.612	0.030				
	CP type	0.977	0.932	-0.007				
	Hamstrings SP	0.013*	0.008*	-0.250	0.539	0.539	20.224	0.001*
	Biceps SP	0.381	0.398	-0.084				
Confounding variables	Hip Adductors SP	0.114	0.132	0.128				
	Epilepsy	0.934	0.607	-0.028				
	Medication	0.542	0.724	-0.020				
	GMFCS	0.000*	0.000*	-0.505				
Predictor Variable	Sleep disturbance	0.001*	0.001*	-0.177	0.565	0.020		

Key: Dependent variable=quality of life; Model 1=before control; model 2=after control; SP=spasticity; CP= cerebral palsy, P=probability level, *= significant.

### Influence of SD on active home-based exercise participation

In the study, there was a significant weak negative correlation between active home-based exercise participation and SD (Rho=-0.36, *p*=0.00). Hierarchical regression analysis revealed that SD (β=-0.225, *p*=0.000) has significant influence on active home-based exercises. Another observation is that medication use (β=-0.395, *p*=0.000) and age (β=-0.209, *p*=0.001) also have significant negative influence on active home-exercise participation even after control as shown in Table 6.

**Table 6:** Influence of SD on Active Home-Based Exercise Participation.

	Variables	P-Value model 1	P-Value model 2	Standardized Coefficient Beta Model 2	R square	R square Change	Repression df	Residual df	F value	P-value
	Age	0.002*	0.001*	-0.209						
	Epilepsy	0.042*	0.101	0.102						
	GMFCS	0.003*	0.097	-0.116						
	Hamstrings	0.971	0.985	-0.002	0.370	0.370	9	199	12.42*	0.000
Confounding	Biceps	0.456	0.461	0.080						
	Hip adductors	0.971	0.901	-0.012						
	Cp type	0.520	0465	0.057						
	Medication	0.000*	0.000*	-0.395						
	Gender	0.741	0.363	0.053						
Predictor variable	Sleep disturbance	0.000*	0.000*	-0.225	0.411	0.041				

Key: Dependent variable = Active home exercise participation, * significant, Model 1=before control; model 2=after control

### Influence of SD on active clinic-based exercise participation

There was a significant weak negative correlation between active clinic-based exercise participation and SD (Rho=-0.36, *p*=0.00). Hierarchical regression analysis revealed that SD has significant influence on active exercise participation in the clinic (β=-0.236, *p*=0.00). It was however observed that medication use (β=-0.389, *p*=0.00), age (β=-0.190, *p*=0.002), and epilepsy (β=-0.135, *p*=0.029) also have significant influence on exercise participation in the clinic even after control (*p*<0.05) as presented in Table 7.

**Table 7:** Influence of SD on Active Clinic-based Exercise Participation.

	Variables	P-Value model 1	P-Value model 2	Standardized Coefficient Beta Model 2	R square	R square Change	Repression DF	Residual DF	F value	P-value
	Age	0.005*	0.002*	-0.190						
	Epilepsy	0.010*	0.029*	0.135						
	GMFCS	0.007*	0.173	-0.095						
	Hamstring	0.912	0.956	0.006	0.370	0.370	9	190	12.41	0.00*
	Biceps	0.689	0.704	0.041						
**Confounding Variables**	Hip adductors	0.912	0.982	0.002						
	Gender	0.935	0.493	0.040						
	Medication	0.000*	0.000*	-0.389						
	Cp type	0.645	0.583	0.43						
**Predictor**	Sleep disturbance	0.000*	0.000*	-0.236	0.415	0.045				

Dependent variable = Active clinic exercise participation *significant, Model 1=before control; model 2=after control

## DISCUSSION

About one-third (31.5%) of the CWCP in this study suffered pathologic SD. This result is similar to the outcomes of previous studies where thirty^4^ and thirty two percent^10^ of pathologic SD have been reported. The prevalence of SD in this study is however higher than the thirteen^7^ and twenty three percent^2^ obtained in other studies probably because majority of the CWCP in the present study have severe motor affectation, which has been reported to be associated with increased level of SD^10, 30^. In this study, CWCP have significantly higher SD when compared with their typically developing siblings who were without any neurological deficit. Inline with the finding of this study, previous studies have also reported that SD was significantly more common among CWCP when compared with typically developing children^2, 4, 6, 7, 8^. SD in CWCP could result from the severity of their motor impairment^2, 11, 10, 30^, musculoskeletal pain^11^, epilepsy^8^, visual impairments^2^, and poor body positioning^11^.

Most of the CWCP in this study, suffered significantly from disorders of excessive somnolence, sleep wake transition, and that of initiation and maintaining sleep. The findings above were in-line with other research reports in which sleep breathing disorders^2, 4, 6, 31^ and disorders of sleep-wake transition^2, 4, 31^, initiating and maintaining sleep^2, 4, 10, 32^, and excessive somnolence^31^ were the frequently reported SD among CWCP.

Excessive somnolence means that many of the CWCP suffered significantly from hypersomnia, difficulty in waking up from sleep or feeling too tired when waking up from sleep^25^. Sleep wake transition disorders means that most of them had sleep bruxism, talked while sleeping or had sleep-related hyperkinesia or hallucinations^25^. The disturbance in the ability to initiate and maintain sleep among participants in this study means that most these children may have suffered short sleep duration, difficulty in falling asleep (long sleep latency), night awakenings or difficulty in sleeping again after waking^25^.

Furthermore, significant association was found between SD and medication use. This implied that the medications used in the treatment of some associated impairments such as spasticity and epilepsy could have significant influence on quality of sleep. Drugs causing excessive daytime sleep make CWCP very drowsy and as such many of them may not participate actively in the therapeutic exercise component of physiotherapy. Also, CWCP who suffered drug-related insomnia the previous night may suffer from daytime weakness and lack of concentration during both clinic and home-based exercises. Studies have reported that SD in CWCP could result from the use of some medications including antiepileptics and anticonvulsants^10, 20, 32^. The observation in this study is that most of the drugs prescribed to CWCP are anticonvulsants, muscle relaxants, or CNS stimulants that have the ability to cause either sleepiness or insomnia. Additionally, this study also found that SD has significant association with presence of epilepsy. This implied that epilepsy is another potential factor that contributed to SD in CWCP. Studies have reported that presence of epilepsy was associated with SD in CWCP^2, 8, 10, 27, 32, 33^.

Linear regression however reveals that the most important factor influencing SD in CWCP in this study was GMF because it has the highest β value. The positive β value implied that as GMF score increases (low level of physical function), SD also increases. As such CWCP with higher GMF values (severe motor impairments) have more pathologic SD. CWCP that have severe motor limitations often experience stiffness, pains, and contractures that could impact negatively on quality of their sleep. This outcome is in-line with the findings of previous studies where it was reported that GMF predicted SD^3, 27^ with children having severe cerebral injury experiencing more SD^34^. Furthermore, studies have also shown that SD was associated with CP of greater severity^2, 8, 10, 11, 30^. Linear regression analysis also revealed that gender has significant influence on SD. The positive β value implied that higher score of gender (female sex was coded = 1 and male sex = 2) leads to higher score of SD. This implied that CWCP who are males, are significantly more prone to have higher SD than females. Another observation in this study is that male children constitute more than half of the children with pathologic SD. Linear regression has also explained that, though medication and epilepsy have contributed to the variance in SD among CWCP, but GMF and male gender were the most important factors leading to the increase in SD. Finally, the model has indicated however that the variables studied have accounted for only 44% (R=0.44) of the variance in SD. This implication of this finding is that, there could be other important variables causing SD in CWCP, which the present study is unable to assess, which could account for the remaining 56% of the variance. Further studies may assess factors such as pain level, severity of musculoskeletal deformities, dysphagia, and severe infections such a pneumonia in relation to SD.

Additionally, it was observed in this study, that the quality of life of CWCP who had pathologic SD was significantly lower than that of CWCP who have insignificant SD. It was also observed that SD had a significant negative relationship with quality of life; implying that, increase in the levels of SD led to significant deterioration of quality of life. This suggested that increase in SD may affect the physical, emotional and cognitive development of CWCP, and this may in turn have negative influence on their quality of life. This is because sleep is essential for physical growth and general health of children^12^. Studies have reported that SD led to deterioration of quality of life^3, 20^. SD such as insomnia and excessive daytime sleepiness have been reported being associated with poor quality of life^3^, while altered pattern of sleep affects the physical and emotional well-being of CWCP^20^.

Although SD was found having significant negative influence on quality of life, this study observe however that, other clinical variables such as GMF levels, hamstring spasticity and age, also have significant influence on quality of life. The GMF which has a negative β-value indicated that higher scores of GMF (low level of physical function) lead to decreased quality of life. The negative β-values of hamstring spasticity and age indicated that high level of spasticity and increase in age significantly leads to deterioration of quality of life. The model of hierarchical regression explained that SD is not the only factor influencing the quality of life of CWCP because other variables such as GMF, spasticity, and age remain significant after being statistically controlled. The outcome above is in-line with the finding from other studies where child related factors such as age^18, 19^, and severity of motor disability^18^, have been found to have negative influence on quality of life in CWCP.

There was a significant weak negative relationship between SD and active participation in home-based exercises. This implied that CWCP having SD tend to participate less actively in home-based exercises. Hierarchical regression analysis revealed that SD has a significant negative influence on active participation in home-based exercises. Another observation from this study was that medication and age also have significant influence on active home-based exercise participation after being controlled statistically. The negative β-value on both medication use and age implies that increased use of medications that interferes with the child’s sleep pattern and older age of CWCP, have negative influence on home-based exercise participation.

There was significant negative relationship between SD and active clinic-based exercise participation. Also hierarchical regression analysis revealed that SD has a significant negative influence on active participation in clinic-based exercise. Another observation from this study was that medication use, epilepsy and increase in child’s age also have significant influence on active participation in clinic-based after these variable have been controlled statistically. This indicated that when assessing variables influencing clinic-based exercise participation among CWCP variables such as SD, the age of the child, presence of epilepsy, and medication use should be taken into considerations.

## CONCLUSION

One-third of the CWCP suffered pathologic SD, which has negative impact on their quality of life and the ability to actively participate in both home and clinic-based exercises. Aside SD, other factors such as child’s age, spasticity level, and severity of motor impairment also affected their quality of life negatively. Enhancing the motor abilities of CWCP may improve their quality of sleep and quality of life.

### What this study adds

1. SD has negative impact on quality of life and the ability of CWCP to actively participate in both home and clinic-based exercise.
2. Besides SD, increase in child’s age, spasticity, and severity of motor impairment also affected quality of life negatively.
3. Apart from SD, increase in child’s age and the use certain medications have negative influence on both active clinic and home-based exercise participation.
4. Physiotherapist can improve quality of sleep in CWCP by enhancing their motor abilities, which is a prerequisite for a better physical and mental functioning. Also, multidisciplinary discus with referring pediatricians may ensure that drugs that caused sedation are not taken close to the time of doing exercise and those drugs causing insomnia could be reviewed.
